# Evaluation of 22G fine-needle aspiration (FNA) versus fine-needle biopsy (FNB) for endoscopic ultrasound-guided sampling of pancreatic lesions: a prospective comparison study

**DOI:** 10.1007/s00464-018-6075-6

**Published:** 2018-02-05

**Authors:** Li Tian, An-Liu Tang, Lei Zhang, Xiao-Wen Liu, Jing-Bo Li, Fen Wang, Shou-Rong Shen, Xiao-Yan Wang

**Affiliations:** 1 Department of Gastroenterology, The Third Xiangya Hospital of Central South University, 138 Tongzipo Road, Changsha, Hunan China; 2 Hunan Key Laboratory of Nonresolving Inflammation and Cancer, Central South University, Changsha, Hunan China; 3 Xiangya School of Medicine, Central South University, Changsha, Hunan China

**Keywords:** Ultrasound-guided fine-needle biopsy, Ultrasound-guided fine-needle aspiration, Pancreatic solid lesion

## Abstract

**Background:**

To compare the diagnostic yield and safety of 22G endoscopic ultrasound-guided fine-needle aspiration (EUS-FNA) and endoscopic ultrasound-guided fine-needle biopsy (EUS-FNB) in the diagnosis of pancreatic solid lesions.

**Methods:**

Between April 2014 and September 2015, 36 patients with pancreatic solid lesions were included for endoscopic ultrasound test. Patients were randomly divided into two groups: EUS-FNA (*n* = 18) and EUS-FNB (*n* = 18). Each nidus was punctured three times (15 ~ 20 insertions for each puncture) with a 22G needle. The core specimens were analyzed, and the diagnostic yields of FNA and FNB were evaluated.

**Results:**

The procedure success rate was 100% with no complications. Cytological and histological examinations found that the diagnostic yield of FNB and FNA were both 83.3%. To get a definitive diagnosis, FNB needed fewer punctures than FNA (1.11 vs. 1.83; *P*  <  0.05).

**Conclusions:**

22G EUS-FNB is a safe and effective way to diagnose pancreatic solid lesions. FNB required a lower number of needle passes to achieve a diagnosis compared with FNA.

Pancreatic cancer is the fourth leading cause of cancer-related deaths in Western countries [1, 2]. Patients who have pancreatic cancer have a poor 5-year survival of only 6%, and 90% of them ultimately die from the disease [3]. Along with the growing demand to treat individuals with precancerous lesions, the need for low-risk investigation, low-morbidity operation, and a minimally invasive approach becomes increasingly pressing [4].

Recently, the standard method for sampling solid pancreatic masses is endoscopic ultrasound-guided fine-needle aspiration (EUS-FNA). Its sensitivity, specificity, and diagnostic accuracy for malignant cytology are 85–95%, 95–98%, and 78–95%, respectively [5, 6]. Despite the success of EUS-FNA, it still has several limitations. First, the availability of a cytopathologist to render on-site diagnosis can affect the diagnostic accuracy [7, 8], and a lower sensitivity of pancreatic mass lesions for EUS-FNA was observed in patients with chronic pancreatitis than in those without chronic pancreatitis [9]. Second, cytology specimens can be of limited value in disease entities diagnosis of which relies on tissue architecture or ancillary studies such as autoimmune pancreatitis [10].

In order to overcome limitations associated with EUS-FNA cytology and to improve diagnostic accuracy, a 19-gauge trucut needle biopsy (EUS-TNB) was designed to procure larger amounts of tissue with conserved architecture that would enable histological analysis [11]. Some initial studies suggested when obtaining biopsy specimens with the EUS-guided true cut biopsy (EUS-TCB) device, TCB has a greater diagnostic accuracy compared with EUS-FNA needles for submucosal mass lesions and lymphoma, and potentially needs fewer needle passes for the diagnosis of solid pancreatic neoplasms [12]. However, it is not feasible to use with the transduodenal approach and has been reported to result in technical failures. The newly developed ProCore needle (Cook Medical), which incorporates reverse bevel technology, has various available sizes and enables the transduodenal approach. In a multicenter, pooled, cohort study, the 19G ProCore needle offers the possibility of obtaining a core sample for histological evaluation in the majority of cases, with an overall diagnostic accuracy of over 85% [13]. The 19G ProCore needle eventually became available in smaller versions that are easier to manipulate (25G and 22G needles) [14]. This endoscopic ultrasound-guided fine-needle biopsy (EUS-FNB) device using a core biopsy needle was developed to improve diagnostic accuracy by simultaneously obtaining cytological aspirates and histologic core samples. The main objective of our prospective experiment is to compare the diagnostic yield of 22G EUS-FNB with 22G EUS-FNA in patients with solid pancreatic lesions and to compare the number of passes needed for diagnosis of FNA and FNB.

## Methods

### Patients

A prospective study of all EUS-guided sampling procedures performed between April 2014 and September 2015 was conducted at The Third Xiangya Hospital of the Central South University. Adult patients were included in the study if they were suspected of having a solid pancreatic mass according to clinical symptoms (pain, jaundice, weight loss, etc.) and/or radiological findings (a solid pancreatic mass exposed by CT and/or MRI and/or EUS). The exclusion criteria were as follows: the presence of predominantly cystic pancreatic lesions (cystic component of more than 50% of the mass on iconography), according to the ESGE guidelines [15], coagulopathy (PT [Quick value] < 60%, PPT > 42 s, and platelets < 60,000/mm^3^), pregnant or lack of pancreatic tissue sampling during the study procedure. Consecutive cases were sampled under the guidance of EUS during the study period, including 36 cases of solid pancreatic lesions. This study was sanctioned by The Third Xiangya Hospital of Central South University. It received approval from the local ethics committee, and signed informed consent was obtained from all patients.

### Procedural technique

The study was prospectively carried out on consecutive cases of solid pancreatic masses, and the patients enrolled were then randomized to undergo 22G EUS-FNB (Cook EchoTip ProCore) using a core biopsy needle or 22G EUS-FNA (Olympus, GF UCT 160) using a standard aspiration needle at the individual tertiary center. All procedures were conducted using Olympus ME1, with patients positioned in the left lateral decubitus position under conscious sedation. All study procedures were performed by a senior endoscopist. FNA or FNB procedures were conducted from duodenum when the masses were located in the pancreatic head or uncinate, while the procedures were conducted from stomach when the masses were located in pancreatic body and tail. Doppler-mode imaging was applied to rule out the presence of any intervening vessels in the needle path.

The same sampling technique was used with both FNA and FNB needles to eliminate technical biases. After the lesion was penetrated by the device, the stylet was removed, and suction was applied by a 5 mL syringe while moving the needle to and fro within the lesion 15 ~ 20 times. Suction was released before removing the needle.

After each pass, tissue material was placed into liquid-based cytology tubes and instantly name tagged and labeled based on the order of each needle pass. Three passes were made using the FNA or FNB. No pathologist was present in the room, and all samples were sent to the pathology department for evaluation.

### Preparation of specimens and measure

The collected material from each pass was simultaneously processed for cytological and histological analysis as follows: the sample placed in CytoLyt solution (a transport medium) was centrifuged and resuspended. A partial resuspended PreservCyt cell fluid sample (20 mL) was smeared onto glass slides which were immediately fixed with 95% ethanol and stained with a Papanicolaou-stain for cytological analysis. For histological analysis, the remaining PreservCyt fluid sample was centrifuged, resuspended, incubated, fixed in formalin, embedded in paraffin, sectioned at 4 µm, and stained with hematoxylin and eosin (H&E). Immunohistochemical studies were performed if needed.

### Cytohistological analysis

Two pathologists who were blinded to the randomization sequence estimated the samples and drew a conclusion about their adequacy and quality. They defined adequacy as the proportion of samples that a final histopathological diagnosis could be made.

### Standard of reference for final diagnosis

A final diagnosis was made using one of the following methods: (i) Definite evidence of malignancy on a surgical sample. (ii) Malignant diagnosis on EUS-FNB or EUS-FNA and clinical/imaging follow-up compatible with malignant disease. (iii) No evidence of malignancy on EUS-FNB or EUS-FNA and on clinical/imaging follow-up of at least 6 months.

### Outcome parameters

The medical records of the patients were revised through standardized data-entry form which included patient demographics, clinical findings, procedural complications, results of follow-up, and pathological findings. The primary outcome was compared with diagnostic yield of FNA and FNB. The secondary objective measures were technical success, sample adequacy, contamination (immediate complications were documented at the time of the procedure, and late complications were documented follow-up at 72 h), complications, the number of passes for diagnosis, and the first pass of diagnosis sensitivity for pancreatic malignancies.

### Statistical analyses

Two-tailed sample size calculation was performed with the Type 2 (b) error set at 0.2 (*χ*^2^ test with corrected continuity) and the Type I error rate (a) set at 0.05 for detecting a difference in diagnostic accuracy between the FNB and FNA groups. *χ*^2^ test or Fisher’s exact test was used to compare categorical data which were expressed as frequencies and proportions, including gender, tumor location, and technical details. Student’s *t* test was used for continuous data like Age and numbers of needle passes required for diagnosis which was reported with mean and standard deviation (SD). The Mann–Whitney U test was used to compare the medians (with interquartile ranges [IQRs] and ranges) of mass size. Sensitivity, specificity, and accuracy values were summarized as exact 95% confidence intervals (CIs). P values were calculated by Fisher’s exact test. The calculation of 95% confidence intervals (CIs) was based on the delta method of logic functional analysis and the asymptotic normality of maximum likelihood estimators. Analyses were performed using SPSS for Windows (version 18.0; SPSS Inc., Chicago, Illinois, USA). Statistical significance was set at a *P* value of < 0.05.

## Results

**Fig. 1:**
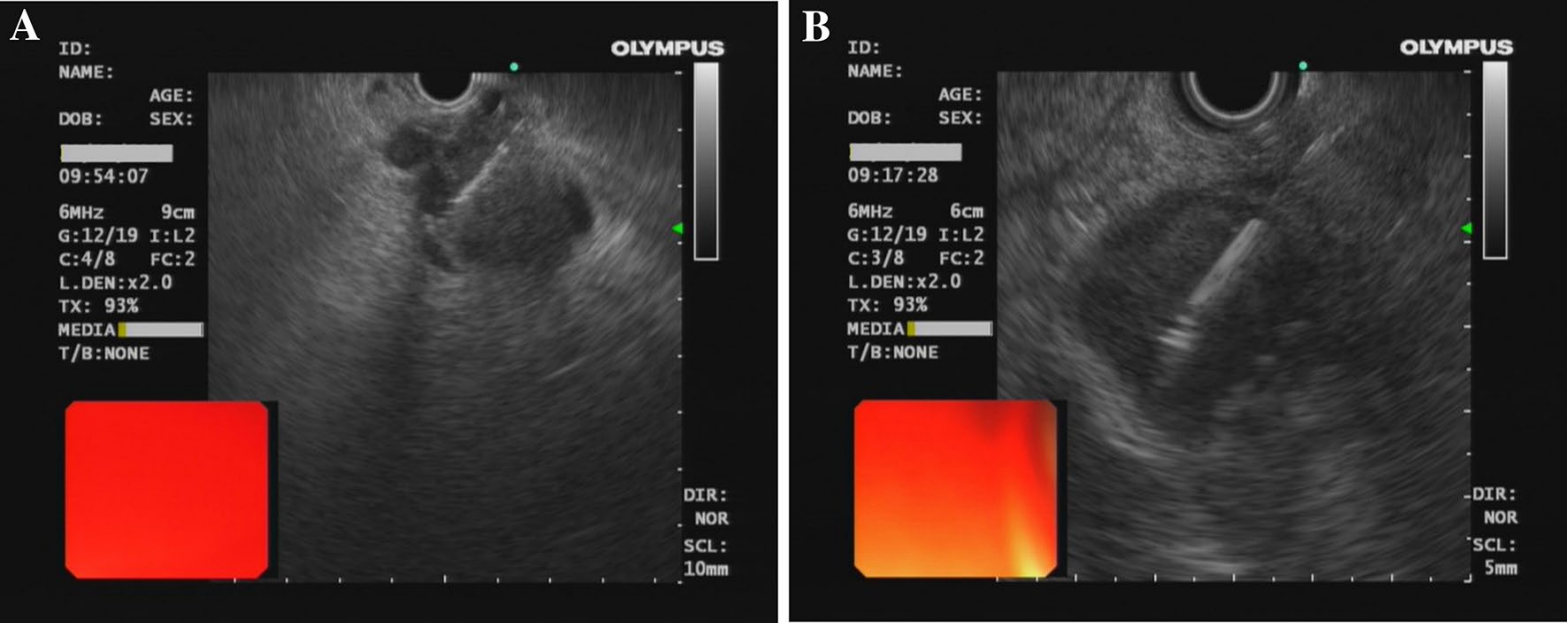
EUS-guided biopsy from pancreatic solid masses using FNA (**A**) and FNB (**B**)

Between April 2014 and September 2015, 36 patients included 23 men and 13 women with a mean age of 59.5 ± 19.5 years (range, 45 ~ 75 years) were screened and all were included. They were randomized to undergo either EUS-FNA group or EUS-FNB group. The demographic details tumor baseline characteristics of the included patients are presented in Table 1. There were no significant difference in patients’ age or gender, or location and size of the tumors, as shown by EUS, between the FNA and FNB group. FNA as well as FNB was technically successful in all patients. Both needles were easily passed through the endoscope, emerged easily from the endoscope, and were clearly visible (Fig. 1).

**Table 1:** Demographic details and pancreatic solid lesions characteristics of 36 patients included in the study

	EUS-FNA	EUS-FNB	*P* value
Mean age (SD), years	61.4 (6.9)	61.2 (9.3)	0.936
Gender, no. (%)			1.000
Female	6 (33.3)	7 (38.9)	
Male	12 (66.7)	11 (61.1)	
Mass location, no. (%)			1.000
Head/uncinate	8 (44.4)	8 (44.4)	
Body/tail	10 (55.6)	10 (55.6)	
Size of mass (mm)			0.304
Median (range)	21.7 (12–36)	19.8 (12–35)	
Interquartile range	16.5	18	
Final diagnosis, no. (%)			
Malignant	9 (50.0)	15 (83.3)	0.075
Benign	9 (50.0)	3 (16.7)	

Results of diagnosis obtained by EUS-FNA/FNB and final diagnoses according to the gold standard are shown in Table 2. In the final diagnosis of the FNA group, 6 of 18 pancreatic mass lesions were pancreatic adenocarcinoma (33.3%), 8 were mass-forming chronic pancreatitis (44.4%), 1 was pancreatic lymphoma (5.6%), 1 was pancreatic tuberculosis (5.6%), 1 was neuroendocrine tumor (5.6%), and 1 was metastasis from gastric cancer (5.6%). But of the patients diagnosed by FNA, 3 misdiagnoses were appeared. 1 patient of metastasis from gastric cancer and 1 patient of mass-forming chronic pancreatitis were misdiagnosed with nonmalignancy, 1 patient of neuroendocrine tumor was misdiagnosed with mass-forming chronic pancreatitis. In the final diagnosis of FNB group, 12 of 18 pancreatic mass lesions were pancreatic adenocarcinoma (66.7%), 1 was mass-forming chronic pancreatitis (5.6%), 2 were pancreatic tuberculosis (11.1%), 1 was neuroendocrine tumor (5.6%), 1 was pancreatic lymphoma (5.6%), and 1 metastasis from gastric cancer (5.6%). However, of the patients diagnosed by FNB, 1 patient of metastasis from gastric cancer and 1 patient of neuroendocrine tumor were misdiagnosed with mass-forming chronic pancreatitis, and 1 patient of metastasis from gastric cancer was misdiagnosed with nonmalignancy. We performed statistical analysis using the results of specimens where a final diagnosis of malignancy was established. Negative predictive value, sensitivity, specificity, and accuracy of EUS-FNB for malignancy (50, 80, 100, and 83%, respectively) were not significantly different from those of EUS-FNA (82, 78, 100, and 89%, respectively; *P* > 0.05). The positive predictive value s of FNA and FNB were 100 and 100%, respectively.

**Table 2:** Results of EUS-FNA/FNB and final diagnoses according to gold standard

EUS-FNA diagnosis	Final diagnosis (gold standard)	EUS-FNB diagnosis	Final diagnosis (gold standard)
Pancreatic adenocarcinoma (*n* = 6)	Pancreatic adenocarcinoma (*n* = 6)	Pancreatic adenocarcinoma (*n* = 12)	Pancreatic adenocarcinoma (*n* = 12)
Mass-forming chronic pancreatitis (*n* = 8)	Mass-forming chronic pancreatitis (*n* = 8)	Mass-forming chronic pancreatitis (*n* = 3)	Mass-forming chronic pancreatitis (*n* = 1)
Pancreatic lymphoma (*n* = 1)	Pancreatic lymphoma (*n* = 1)	Pancreatic tuberculosis (*n* = 2)	Pancreatic tuberculosis (*n* = 2)
Pancreatic tuberculosis(*n* = 1)	Pancreatic tuberculosis (*n* = 1)	Nonmalignancy (*n* = 1)	Neuroendocrine tumor (*n* = 1)
Nonmalignancy (*n* = 2)	Neuroendocrine tumor (*n* = 1)		Pancreatic lymphoma (*n* = 1)
	Metastasis from gastric cancer (*n* = 1)		Metastasis from gastric cancer (*n* = 1)

**Fig. 2:**
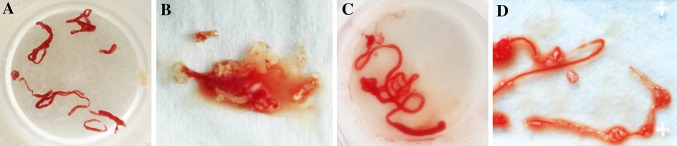
Tissue core obtained using the 22G fine-needle biopsy needle by EUS-FNA (**A**) and FNB (**B**)

**Fig. 3:**
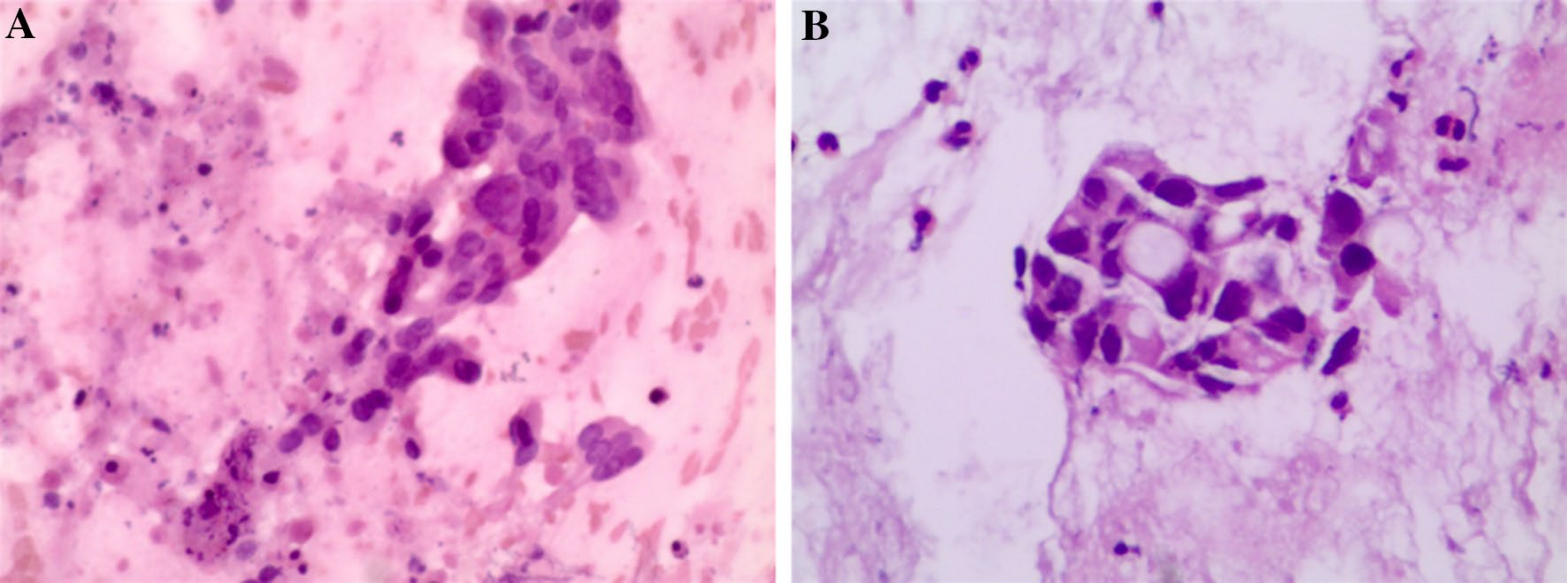
Histological evaluation of specimens obtained (hematoxylin & eosin [H&E] staining). **A** pancreatic ductal adenocarcinoma, sample obtained using the new 22G fine-needle biopsy needle by EUS-FNA. **B** pancreatic ductal adenocarcinoma, sample obtained using standard fine-needle aspiration needle by EUS-FNB

Table 3 summarizes the results relevant to the primary and secondary endpoints. No technical failures were observed in either group. No complications occurred in both groups. Cytological and histological examination found that the diagnostic yields of FNB and FNA were both 83.3%. No difference was found in diagnostic yield between the two techniques. The important thing is, the number of needle passes for diagnosis of FNB (1.11 ± 0.83) was significantly lower than that for the FNA (1.83 ± 1.25; *P* = 0.049). On subgroup analysis of 24 patients with malignant lesions, the number of patients in whom a malignancy was diagnosed by a combination of cytological and histological analysis on the first pass was significantly greater in the FNB group than that in the FNA group (80.00 vs. 66.67%; *P* = 0.019). Adequate material for pathological analysis was obtained in 15/18 FNB samples and also in 15/18 FNA samples, showing no difference between the two groups. The overall quality of the samples obtained from patients with either needle is shown with the average assessments of one expert pathologist (Figs. 2, 3). The FNB group gained a higher average score; however, the difference was not statistically significant.

**Table 3:** Technical characteristics and outcomes for EUS-FNA/FNB

Characteristic	FNA (*n* = 18)	FNB (*n* = 18)	*P* value
Technical success, no. (%)	18 (100)	18 (100)	
Diagnostic yield, no. (%)	15 (83.3)	15 (83.3)	
No. of passes for diagnosis, mean (SD)	1.83 (1.25)	1.11 (0.83)	0.049
No. of diagnoses of malignancy made in pass 1, sensitivity (%)	6 (66.67)	12 (80.00)	0.019
Complications, no. (%)	0 (0)	0 (0)	

## Discussion

In the present study, we found that the use of the 22G FNB needle was technically viable, safe, and efficient, which was comparable to the use of a standard FNA needle in patients with pancreatic masses. The total diagnostic yield of FNB was equivalent to that of a standard FNA. The adequacy of the specimen obtained by the FNB needle was comparable to that of the FNA needle. The technical performances were similar to each other. However, fewer passes were required to obtain a diagnosis by FNB than were required by FNA.

The pathological diagnosis of solid pancreatic masses is important to ensure that suitable therapy is given and the optimum prognosis is achieved [16, 17]. EUS-guided FNA of solid malignancies accessible from the upper alimentary tract is a safe, with highly diagnostic accuracy (> 90%) when a rapid on-site pathologist is available [18]. However, the diagnostic accuracy of EUS-FNA with cytology alone is insufficient to verify cellular arrangement and tissue architecture [19], which limited sample of tissue to be obtained, thereby potentially reducing diagnostic performance. To improve sampling and diagnostic accuracy, a number of measures have been offered [20, 21]. A real-time sample adequacy evaluation from an onsite cytopathologist has been reported to increase the yield of samples by 10 ~ 15% [22]. Nevertheless, because of increased expenses and a longer procedure time, on-site cytology is not available in all facilities. With the continued interest in the development of an EUS-guided platform, a 19G FNA needle and the Quick-Core or Trucut instrument appeared [10, 23, 24], but all of these approaches have been met with technical limitations or a lack of demonstrated efficacy, particularly in the transduodenal position. In recent years, an EUS-FNB device using a core biopsy needle was developed to improve diagnostic accuracy by simultaneously obtaining cytological aspirates and histological core samples [25], which may have comparable diagnostic yield to traditional FNA for pancreatic solid lesions in both the 19G and 22G diameters [13, 25].

In our study, we compared FNA and FNB in many respects. The results of the experiment indicate that both FNA and FNB are available and safe during operation. Our results showed that there were no procedure-related complications among the 36 patients, some of whom complained with obstructive jaundice, abdominal pain, or body weight loss before operation. Safety of EUS-FNA has been reported, and complication rates range between 0.5 and 3% [13, 25–27]. With respect to technical performance, Iglesias-Garcia et al. [13] evaluated the 19G FNB in 114 lesions of 109 patients; two technical failures (5.7%) occurred through duodenal approach. Furthermore, the needle emerged from the endoscope with difficulty in 18% of cases and with great difficulty in 1%. The difficulty encountered was attributed to a curved position of the endoscope in the duodenum. In contrast, we experienced no technical difficulties when performing biopsies with our 22G FNA and FNB and no technical failures. In all our cases, the needle emerged easily from the endoscope, irrespective of the location of the endoscope in the gastrointestinal tract, which is similar to the study of Hucl et al. [27].

We found that the adequacy of samples obtained by FNB and FNA showed no statistically significant difference. In a prior randomized, retrospective study comparing a 22G beveled needle with a standard 22G FNA needle, the researchers demonstrated that fewer passes may be required with the beveled needle to achieve adequate sample compared to the standard needle; however, no meaningful difference in case duration was evident between the two needle types [28]. Similar findings were seen in another prospective study comparing the yields of 22G conventional FNA and 22G FNB [25]. The randomized, single group, prospective study by Mavrogenis et al. assesses the diagnostic yield of the 25G ProCore versus the 22G standard cytology needle in pancreatic mass lesions and lymphadenopathy adjacent to the upper gastrointestinal tract. They found no significant differences between the two needles in terms of diagnostic yield [29]. Several factors might have contributed to the lower than expected diagnostic accuracy of the FNB device. Further prospective research with a larger population of patients with solid pancreatic masses is still needed. In cases sampled by FNA or FNB, Witt et al. [28] assessed the average number of needle passes required to procure each specimen and noted that the mean number of needle passes was significantly lower for the FNB cohort, which is comparable to our study. The fewer number of needle passes means the shorter procedure duration which is considered to be an advantage, reducing the need for anesthesia, as well as cost and rates of complications. There was no significant difference in the average number of needle passes per FNA or FNB specimen in the study of Bang et al. [25]. However, in their study, immediate cytological evaluation was available, and this might potentially reduce the number of needle passes of FNA. Data on the performance of the 22G ProCore needle suggest a 78.7% first-pass sensitivity for pancreatic malignancy, which was higher than the 32.4% first-pass sensitivity achieved with the FNA needle. In the study of Mavrogenis et al. [29], both the 22G standard needle and the 22G ProCore needle had a slightly better single-pass performance. Based on these results, the higher single-pass diagnostic yield of the FNB device may reduce the number of passes required to obtain adequate specimens, which would be especially helpful in hospitals lacking on-site cytology.

One of the limitation of the study is the small number of patients included. The fewer numbers of malignancy in FNA group also introduces bias in the analysis. Therefore, a larger patient samples are needed to confirm our results. In addition, it was not possible to blind the endoscopist to the type of device used during the procedures, but it would not be a major limitation because the pathologists were blinded to the randomization sequence and the type of device used for tissue sampling.

## Conclusion

EUS-guided FNA and FNB are convenient, safe procedure that has the ability to provide diagnoses with good sensitivities and specificities for the evaluation and diagnosis of lesions within the gastrointestinal tract and adjacent organs. Our study showed that compared to the standard FNA needles of the same gauge, the 22G reverse-beveled FNB needles required a lower number of needle passes to achieve a diagnosis. The total diagnostic yield, quality of sampling technical performance, and complication rates of the new 22G FNB needle were comparable with the 22G FNA needle.

## Abbreviations

EUS: Endoscopic ultrasound
EUS-FNA: Endoscopic ultrasound-guided fine-needle aspiration
EUS-FNB: Endoscopic ultrasound-guided fine-needle biopsy
TNB: Trucut needle biopsy
TCB: True cut biopsy

## References

[1] Maitra A, Hruban RH (2008). Pancreatic cancer. Annu Rev Pathol.

[2] Cui X, Zhang Y, Yang J, Sun X, Hagan JP, Guha S, Li M (2014). ZIP4 confers resistance to zinc deficiency-induced apoptosis in pancreatic cancer. Cell Cycle.

[3] Pietryga JA, Morgan DE (2015). Imaging preoperatively for pancreatic adenocarcinoma. J Gastrointest Oncol.

[4] Del Chiaro M, Segersvard R, Lohr M, Verbeke C (2014). Early detection and prevention of pancreatic cancer: is it really possible today?. World J Gastroenterol.

[5] Ngamruengphong S, Li F, Zhou Y, Chak A, Cooper GS, Das A (2010). EUS and survival in patients with pancreatic cancer: a population-based study. Gastrointest Endosc.

[6] Othman MO, Wallace MB (2012). The role of endoscopic ultrasonography in the diagnosis and management of pancreatic cancer. Gastroenterol Clin North Am.

[7] Erickson RA, Sayage-Rabie L, Beissner RS (2000). Factors predicting the number of EUS-guided fine-needle passes for diagnosis of pancreatic malignancies. Gastrointest Endosc.

[8] Song TJ, Kim JH, Lee SS, Eum JB, Moon SH, Park do H, Seo DW, Lee SK, Jang SJ, Yun SC, Kim MH (2010). The prospective randomized, controlled trial of endoscopic ultrasound-guided fine-needle aspiration using 22G and 19G aspiration needles for solid pancreatic or peripancreatic masses. Am J Gastroenterol.

[9] Varadarajulu S, Tamhane A, Eloubeidi MA (2005). Yield of EUS-guided FNA of pancreatic masses in the presence or the absence of chronic pancreatitis. Gastrointest Endosc.

[10] Mizuno N, Bhatia V, Hosoda W, Sawaki A, Hoki N, Hara K, Takagi T, Ko SB, Yatabe Y, Goto H, Yamao K (2009). Histological diagnosis of autoimmune pancreatitis using EUS-guided trucut biopsy: a comparison study with EUS-FNA. J Gastroenterol.

[11] Levy MJ (2007). Endoscopic ultrasound-guided trucut biopsy of the pancreas: prospects and problems. Pancreatology.

[12] Levy MJ, Wiersema MJ (2005). EUS-guided Trucut biopsy. Gastrointest Endosc.

[13] Iglesias-Garcia J, Poley JW, Larghi A, Giovannini M, Petrone MC, Abdulkader I, Monges G, Costamagna G, Arcidiacono P, Biermann K, Rindi G, Bories E, Dogloni C, Bruno M, Dominguez-Munoz JE (2011). Feasibility and yield of a new EUS histology needle: results from a multicenter, pooled, cohort study. Gastrointest Endosc.

[14] Lee YN, Moon JH, Kim HK, Choi HJ, Choi MH, Kim DC, Lee TH, Cha SW, Cho YD, Park SH (2014). Core biopsy needle versus standard aspiration needle for endoscopic ultrasound-guided sampling of solid pancreatic masses: a randomized parallel-group study. Endoscopy.

[15] Dumonceau JM, Polkowski M, Larghi A, Vilmann P, Giovannini M, Frossard JL, Heresbach D, Pujol B, Fernandez-Esparrach G, Vazquez-Sequeiros E, Gines A (2011). Indications, results, and clinical impact of endoscopic ultrasound (EUS)-guided sampling in gastroenterology: European Society of Gastrointestinal Endoscopy (ESGE) Clinical Guideline. Endoscopy.

[16] Iglesias-Garcia J, Larino-Noia J, Abdulkader I, Forteza J, Dominguez-Munoz JE (2010). Quantitative endoscopic ultrasound elastography: an accurate method for the differentiation of solid pancreatic masses. Gastroenterology.

[17] Erickson RA, Garza AA (2000). Impact of endoscopic ultrasound on the management and outcome of pancreatic carcinoma. Am J Gastroenterol.

[18] Klapman JB, Logrono R, Dye CE, Waxman I (2003). Clinical impact of on-site cytopathology interpretation on endoscopic ultrasound-guided fine needle aspiration. Am J Gastroenterol.

[19] Wahnschaffe U, Ullrich R, Mayerle J, Lerch MM, Zeitz M, Faiss S (2009). EUS-guided Trucut needle biopsies as first-line diagnostic method for patients with intestinal or extraintestinal mass lesions. Surg Endosc.

[20] Gimeno-Garcia AZ, Elwassief A (2012). How to improve the success of endoscopic ultrasound guided fine needle aspiration cytology in the diagnosis of pancreatic lesions. J Interv Gastroenterol.

[21] Polkowski M, Larghi A, Weynand B, Boustiere C, Giovannini M, Pujol B, Dumonceau JM (2012). Learning, techniques, and complications of endoscopic ultrasound (EUS)-guided sampling in gastroenterology: European Society of Gastrointestinal Endoscopy (ESGE) Technical Guideline. Endoscopy.

[22] Iglesias-Garcia J, Dominguez-Munoz JE, Abdulkader I, Larino-Noia J, Eugenyeva E, Lozano-Leon A, Forteza-Vila J (2011). Influence of on-site cytopathology evaluation on the diagnostic accuracy of endoscopic ultrasound-guided fine needle aspiration (EUS-FNA) of solid pancreatic masses. Am J Gastroenterol.

[23] Thomas T, Kaye PV, Ragunath K, Aithal G (2009). Efficacy, safety, and predictive factors for a positive yield of EUS-guided Trucut biopsy: a large tertiary referral center experience. Am J Gastroenterol.

[24] Larghi A, Verna EC, Ricci R, Seerden TC, Galasso D, Carnuccio A, Uchida N, Rindi G, Costamagna G (2011). EUS-guided fine-needle tissue acquisition by using a 19-gauge needle in a selected patient population: a prospective study. Gastrointest Endosc.

[25] Bang JY, Hebert-Magee S, Trevino J, Ramesh J, Varadarajulu S (2012). Randomized trial comparing the 22-gauge aspiration and 22-gauge biopsy needles for EUS-guided sampling of solid pancreatic mass lesions. Gastrointest Endosc.

[26] Tharian B, Tsiopoulos F, George N, Pietro SD, Attili F, Larghi A (2013). Endoscopic ultrasound fine needle aspiration: technique and applications in clinical practice. World J Gastrointest Endosc.

[27] Hucl T, Wee E, Anuradha S, Gupta R, Ramchandani M, Rakesh K, Shrestha R, Reddy DN, Lakhtakia S (2013). Feasibility and efficiency of a new 22G core needle: a prospective comparison study. Endoscopy.

[28] Witt BL, Adler DG, Hilden K, Layfield LJ (2013). A comparative needle study: EUS-FNA procedures using the HD ProCore(™) and EchoTip(®) 22-gauge needle types. Diagn Cytopathol.

[29] Mavrogenis G, Weynand B, Sibille A, Hassaini H, Deprez P, Gillain C, Warzee P (2015). 25-gauge histology needle versus 22-gauge cytology needle in endoscopic ultrasonography-guided sampling of pancreatic lesions and lymphadenopathy. Endosc Int Open.

